# Transferability of a 10-week remotely delivered Virtual Physical Activity Seated Exercise (V-PASE) program on post-stroke functional mobility: study protocol for a multisite randomized controlled trial

**DOI:** 10.1186/s13063-026-09523-8

**Published:** 2026-02-06

**Authors:** Paul Mackie, Maureen C. Ashe, Ruth Barclay, Mark T. Bayley, Sarah J. Donkers, Jamie L. Fleet, W. Ben Mortenson, Sue Peters, Courtney L. Pollock, Sepideh Pooyania, Adria Quigley, Brodie M. Sakakibara, Amy Schneeberg, Lisa Sheehy, Sally Stelling, Jennifer Yao, Janice J. Eng

**Affiliations:** 1 Department of Physical Therapy, University of British Columbia, 2177 Wesbrook Mall, Vancouver, BC V6T 1Z3 Canada; 2 Rehabilitation Research Program, Vancouver Coastal Health Research Institute, Vancouver, BC Canada; 3 Centre for Aging SMART, Vancouver Coastal Health Research Institute, Vancouver, BC Canada; 4 Department of Family Practice, University of British Columbia, Vancouver, BC Canada; 5 Department of Physical Therapy, College of Rehabilitation Science, University of Manitoba, Winnipeg, MB Canada; 6 Division of Physical Medicine and Rehabilitation, Department of Medicine, University of Toronto, Toronto, ON Canada; 7 School of Rehabilitation Science, College of Medicine, University of Saskatchewan, Saskatoon, SK Canada; 8 Lawson Research Institute, St. Joseph’s Health Care, London, ON Canada; 9 Physical Medicine and Rehabilitation, Schulich School of Medicine and Dentistry, University of Western Ontario, London, ON Canada; 10 Department of Occupational Science and Occupational Therapy, University of British Columbia, Vancouver, BC Canada; 11 School of Physical Therapy, Western University, London, ON Canada; 12 Riverview Health Centre, Winnipeg, MB Canada; 13 School of Physiotherapy, Dalhousie University, Halifax, NS Canada; 14 Nova Scotia Health Authority, Halifax, NS Canada; 15 Centre for Chronic Disease Prevention and Management, The University of British Columbia Okanagan, Kelowna, BC Canada; 16 Bruyère Health Research Institute, Ottawa, ON Canada; 17 BC Brain Wellness Program, Djavad Mowafaghian Centre for Brain Health, Vancouver, BC Canada; 18 Department of Physical Therapy, Physical Therapy and Research Clinic, University of British Columbia, Vancouver, BC Canada; 19 Division of Physical Medicine and Rehabilitation, University of British Columbia, Vancouver, BC Canada

**Keywords:** Stroke, Videoconferencing, Rehabilitation, Mobility, Balance, Seated exercise

## Abstract

**Background:**

Seated exercises may reduce the need for in-person support during home-based exercise programs in people with balance impairments. However, it is uncertain if these exercises can transfer to improved lower extremity function and mobility. Thus, the objective is to investigate the effects of a remotely delivered 10-week seated exercise intervention on functional mobility, compared with control, in individuals living with a chronic stroke who have balance impairments.

**Methods:**

The study is a multi-site, assessor blinded, randomized controlled trial that will recruit across five provinces in Canada using the CanStroke Recovery Trials platform. A total of 100 adults living with a chronic stroke (≥ 6 months post-stroke) and mobility impairment (using a walking aid) will be recruited. Participants will be randomized (1:1) to the 10-week Virtual Physical Activity Seated Exercise (V-PASE) or control group. All exercise sessions will be delivered one-on-one through videoconferencing by a trained instructor. Sessions will be 60 min in duration and completed 3 times/week at a moderate intensity (40%–60% Heart Rate Reserve). The primary outcome measure is the 30s Sit-To-Stand score at the end of the 10-week intervention. Secondary outcome measures will be mobility, balance, quality of life, stroke-related quality of life, cognition, fatigue, anxiety, depression, and blood profiles (glucose and lipids).

**Discussion:**

Exercises completed in a chair have the potential to transfer to improved functional mobility in people with balance impairments, such as individuals with stroke. The stability of the seated position may improve safety during home-based exercises and thus increase participation.

**Trial registration:**

ClinicalTrials.gov NCT05724823. Registered on February 13th, 2023.

**Supplementary Information:**

The online version contains supplementary material available at 10.1186/s13063-026-09523-8.

## Background

One in four people aged over 25 will experience a stroke over their lifetime [1] and up to 46% will live with a stroke-related disability [2]. Disability manifests as a variety of physical and psychological impairments, with impaired mobility the most reported (58% of people with stroke) long-term consequence after stroke [3]. Improving mobility is an important priority for individuals living with stroke [4, 5]. However, functional mobility (balance and walking ability) can deteriorate over time in people with moderate severity strokes [6] and could result in reduced independence in daily activities [7] and an increased risk of falls [8].

Regular physical activity can improve mobility [9] and modifiable risk factors (cardiovascular and metabolic risk factors) for recurrent stroke [10]. Interventions that incorporate circuit training, aerobic training, or multimodal training have been found to improve balance and mobility outcomes in people with stroke [11–13]. However, people with stroke often report impaired function, fear of falling, and limited access to services and community programs as barriers to engaging in physical activity [14–16]. This reinforces the need for safe home-based programs that can be delivered remotely to increase physical activity engagement and improve mobility after stroke.

Home-based exercise programs have been found to produce similar improvements in mobility and balance in people with acute and chronic stroke, when compared with exercises delivered at hospital or community centres [17]. When delivered remotely through videoconferencing, home-based exercise interventions are also reported to improve mobility [18] and perceived fitness and function [19] after stroke. Galloway et al. [19] reported high feasibility of a remotely delivered exercise intervention, with 95% of participants reporting high usability and satisfaction. However, a survey of physiotherapists (*n* = 75) working in predominately outpatient or community settings found 70% (*n* = 53) believed they could not treat mobility or balance effectively over videoconferencing, and 53% believed that participant safety was a barrier to using videoconferencing to provide treatment [20]. In addition, a barrier to participation in remotely delivered exercise trials is the typical requirement for someone to be at home to assist, with up to 39% of stroke survivors being ineligible due to the lack of available in-person assistance [19, 21]. Thus, improving the stability of the exercising participant through seated exercises may be a safer alternative to traditional walking exercises when delivered remotely, and remove the need for in-person physical assistance.

In-person seated exercise programs have been shown to improve balance and mobility outcomes in people living with stroke [22]. In a recent case report conducted by our team, we found that two weeks of seated exercises delivered through videoconferencing resulted in clinically meaningful changes in balance, lower extremity strength, walking speed, and cardiorespiratory fitness in a person living with a chronic stroke [23]. In addition, the participant experienced no adverse events or falls. Our case report highlights the potential for seated exercises to be a safe alternative to traditional standing and walking exercise for home-based programs and provides the basis for this clinical trial. Thus, the objective is to determine the effects of a remotely delivered 10-week seated exercise intervention on functional standing and walking mobility in people with a chronic stroke and mobility impairment.

## Methods

### Study design

**Fig. 1:**
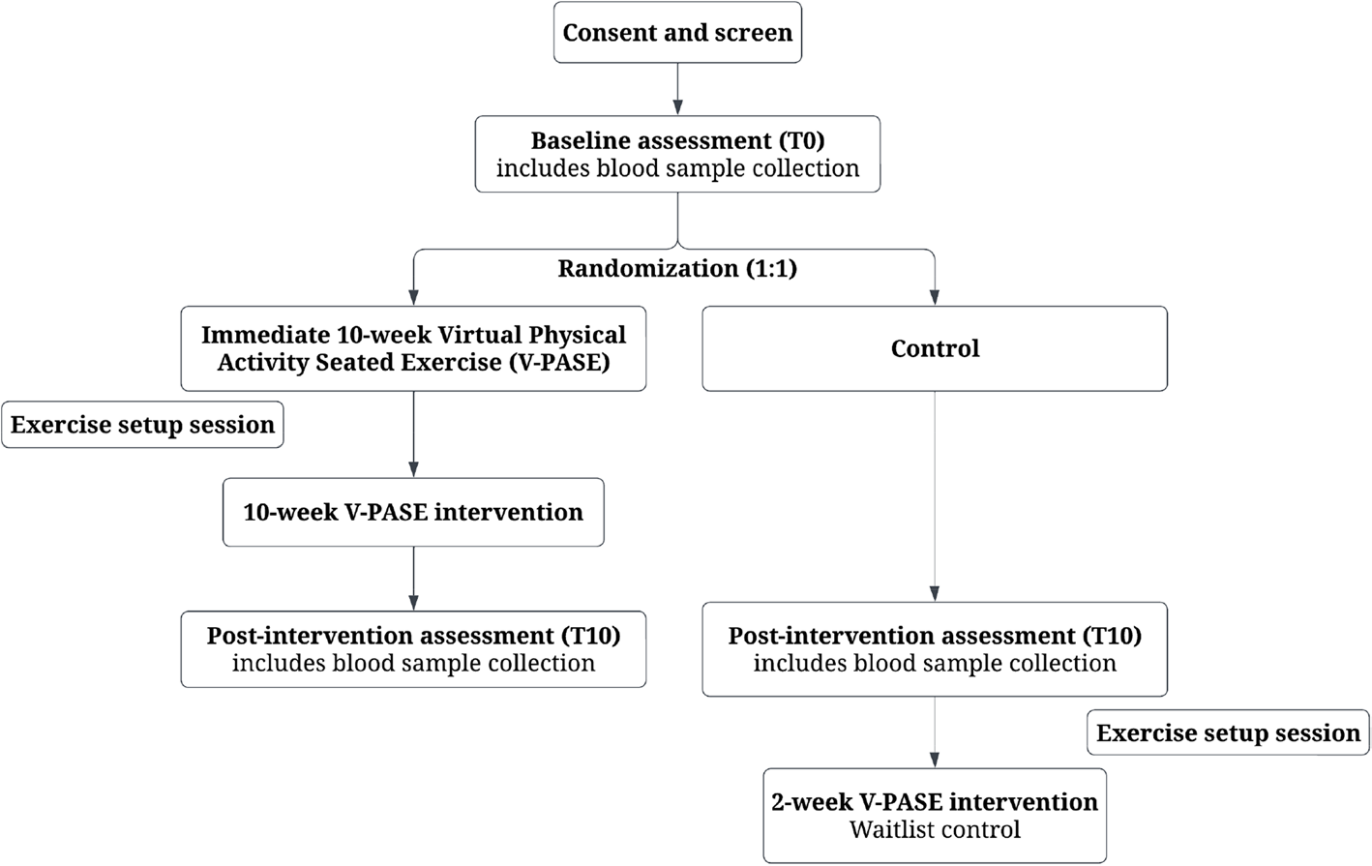
Flow diagram of assessor-blinded, multi-site randomized controlled trial

This study is an assessor-blinded, parallel-group, multi-site randomized controlled trial (Fig. 1). Participants will be randomized 1:1 to a 10-week Virtual Physical Activity Seated Exercise group (V-PASE) or a control group continuing with usual care (Control) after completion of the baseline assessment. The study will be delivered in real-time through videoconferencing services. Assessors will be blinded to the group assignments. The study protocol (Version 9, April 5th, 2024) is registered with clinicaltrials.gov (NCT05724823) and adheres to the Standard Protocol Items: Recommendations for Intervention Trials [24] and CONSORT [25] guidelines.

### Study setting

The study will recruit community-dwelling people living with chronic stroke from sites in British Columbia, Saskatchewan, Manitoba, Ontario, and Nova Scotia that are associated with the Canstroke Recovery Trials Platform (https://canadianstroke.ca/). Recruitment will be conducted through affiliated hospitals and outpatient centres of site investigators and through local community and stroke recovery programs. The central research team at GF Strong Rehabilitation Centre (Vancouver, BC) will support individual sites on the study setup (including ethics), training, and processes. However, each site will independently recruit, consent, screen, enrol, schedule (assessments, exercise sessions, and blood work), input and manage data, and follow up participants. The central team will train staff at each site on the study protocols and will conduct fidelity assessments at regular intervals during the study.

### Population

The project will recruit community-dwelling adults (where adults are defined by the province) living with a chronic stroke (≥ 6 months). People with stroke will be included if they (1) have a mobility impairment (i.e., uses a walking aid in outdoor or large indoor spaces), (2) can stand up from a sitting position (with or without support or assistive devices), (3) have access to the internet and a device with a video camera, and (4) are able to communicate in English. Exceptions will be made to the English language criteria if assessors and instructors can translate for the participant.

People with stroke will be excluded if they (1) are participating in a formal exercise program or engaging in physical activity that exceeds 150 minutes/week moderate-intensity physical activity, (2) are enrolled in another exercise trial or rehabilitation services (e.g., lower extremity physiotherapy) that may affect the outcomes of the study, (3), have severe loss of hearing, speech, or vision, or (4) have a serious comorbid condition or have been diagnosed with a condition that could affect participation (e.g., leg amputation, Parkinson’s disease, active cancer). In addition, a helper (e.g., caregiver, family member, or friend) is required to attend in-person during outcome assessments only, but they are not required during exercise sessions.

### Randomization

Randomization will be completed through an online third-party service (Diamind Solutions) following completion of the baseline assessment. Enrolled participants will be randomized within site, by coordinators, to the V-PASE or control group. Group allocation will be blinded for assessors only, and assessors will be instructed to refrain from asking the participant about study details. Randomization will be conducted using the Minimum Sufficient Balance (MSB) [26] algorithm that ensures balanced allocation across specific stratification factors. Participants will be stratified based on site, age (continuous variable; years), sex (categorical; male, female), time post-stroke (continuous; months), Modified Rankin Scale (continuous; 0–5), Fatigue Assessment Scale (continuous; 10–50), and walking speed calculated from the Timed Up and Go (continuous; cm/s).

The MSB algorithm will detect any imbalances in allocations across groups and will adjust the probability of assignment according to the following parameters. The first 10 participants will have a 50% (50/50) probability of being assigned to the V-PASE group or the control group. If an imbalance is detected in the next 80% of participants, the algorithm will adjust the probability assignment from 50/50 to 70/30. In the final 20% of participants, the probability will be adjusted to 90/10 to correct any imbalances.

### Initial setup session and safety considerations

A 60-minute setup session will be completed by the trained instructor with the participant via videoconferencing services prior to starting the seated exercise intervention. Prior to the setup session, participants will be mailed an equipment package that contains one Fitbit Versa 4 smartwatch (Versa 4, Fitbit, Inc, Canada), a Samsung Galaxy Tab A7 tablet, two adjustable weighted cuffs (1 lb each), a V-PASE exercise manual containing pictures and descriptions of the exercises, and a Fitbit Versa 4 and Samsung Galaxy Tab A7 user manual. During the setup session, the instructor will show the participant how to use the equipment, will conduct a safety check of the participant's environment (visual), and will complete a demonstration of the exercises. The instructor will perform each exercise with the participant and ensure exercises are performed safely and correctly. The instructor can modify exercises if a participant reports any discomfort or pain during an exercise.

The participant will be instructed to wear the Fitbit watch on the non-paretic wrist during the setup session and exercise sessions. In the setup session, the instructor will ask the participant to report their current heart rate after 10 minutes of seated rest. After each session, the participant will be asked to sync the Fitbit watch with the Fitbit app on the Samsung Tablet provided so that a data accuracy check can be completed by the site coordinator.

### Intervention

Seated exercises will be delivered one-on-one remotely through videoconferencing services three times a week by a trained instructor with a degree in kinesiology, physical therapy, or occupational therapy and experience in delivering exercise interventions. The intervention group (V-PASE) will complete a total of 30 sessions, and the control group will continue with usual care. Usual care may involve participating in recreational programs or physical activity that does not exceed 150 minutes per week of moderate-intensity physical activity or upper extremity rehabilitation. The control group will receive a 2-week (6 sessions) seated exercise intervention on completion of the post-intervention assessment to facilitate retention in the study.

Exercise sessions will be 60 minutes in duration and will include a 5-minute warm-up, 50 minutes of structured exercises, and a 5-minute cool-down. Twelve exercises (upper body, lower body, core) will be completed during the 50-minute structured exercise component that will focus on aerobic fitness, weight-bearing, strength training, agility, and coordination. Details of the twelve exercises are listed in Supplementary Table 1 and have also been described and published elsewhere [23].

Instructors will modify exercises according to participant function and will progress exercises by increasing the number of repetitions (e.g., 10 to 30 repetitions), sets (e.g., 3 to 5 sets), duration (e.g., 1 to 5 minutes), speed of movement, using weighted cuffs (1 lb each), and transitioning from mini-lifts to full sit-to-stands. Exercises will be completed in a sturdy chair and participants will be instructed to work at a moderate intensity of 40%–60% Heart Rate Reserve (HRR) [27]. To ensure a moderate intensity is maintained during sessions, priority will be placed on aerobic and lower extremity exercises as demonstrated in our case-report [23]. The instructor will ask the participant to report their heart rate (using the Fitbit Versa 4 watch) and perceived physical exertion (using the 0–10 Rating of Perceived exertion scale [RPE, 0 = rest, 10 = maximal]) at regular intervals in each session to ensure target intensity is achieved. At-home support from a helper (e.g., caregiver, family member) is not required during seated exercise sessions. However, instructors will conduct a safety check at the start of each session confirming the participant’s health, contact details, and location.

### Outcomes

Outcomes will be collected remotely through videoconferencing at baseline (T0) and immediately post-intervention (T10) (Table 1). Assessments will be conducted by a trained assessor who has a degree or qualification in physical therapy or occupational therapy. Assessors will be blinded to the participant group.

**Table 1:** Schedule of baseline and post-intervention assessments

Timepoint	Baseline (T0)		Post-intervention (T10)	
Group	V-PASE	Control	V-PASE	Control
Demographics	x	x		
Primary outcome				
Modified 30 s Sit-To-Stand test	x	x	x	x
Secondary outcomes				
Fatigue Severity Scale	x	x	x	x
Activities-specific Balance Confidence Scale	x	x	x	x
Modified Fugl-Meyer – lower extremity	x	x	x	x
Tandem stance	x	x	x	x
Stroke Impact Scale	x	x	x	x
Timed Up and Go	x	x	x	x
EQ-5D-5L	x	x	x	x
PHQ-4	x	x	x	x
MoCA-BLIND	x	x	x	x
Blood profiles *HbA1c, fasting glucose, cholesterol (total, HDL, LDL), lipid, triglycerides*	x	x	x	x

*V-PASE* Virtual Physical Activity Seated Exercise, *EQ-5D-5L* EuroQol-5D-5L, *PHQ-4* Patient Health Questionnaire, *MoCA-BLIND* Montreal Cognitive Assessment BLIND, *HbA1c* hemoglobin A1c, *HDL* high-density lipoprotein, *LDL* low-density lipoprotein

Participant demographics will be collected at baseline (T0) and include age, sex, gender (using the Stanford Gender Questionnaire [28]), employment status, living arrangements, type of walking aid used, comorbidities (Functional comorbidity index [29]), medication, stroke profile (date, number of strokes, type of stroke, side affected, Modified Rankin Scale [30], adapted National Institute of Health Stroke Scale [motor arm, motor leg, limb ataxia, best language, dysarthria] [31, 32]), and fatigue (Fatigue Assessment Scale [33]).

### Primary outcome

The primary outcome will be functional mobility using the modified 30 second Sit-To-Stand (STS) [34] score immediately post-intervention (T10). The modified 30 s STS test allows participants the opportunity to use their arms if they are unable to complete a STS unassisted, providing a more appropriate assessment of lower extremity strength performance in lower functioning individuals [34]. Participants will be seated on a standard chair with armrests and will be asked to complete as many STS as possible within 30 s. One practice trial will be allowed prior to the assessment, and participants will be encouraged to perform the modified 30 s STS test without the use of arms. If a participant uses their arms during the modified 30 s STS test at baseline (T0), then at T10 they will first attempt a modified 30 s STS test without using arms, then perform the modified 30 s STS with use of their arms.

### Secondary outcomes

Balance and mobility will be measured by the Timed Up and Go (TUG) [35, 36] where participants will be asked to stand up from an armed chair, walk 3 m at a comfortable pace, and then return to the chair. One practice TUG will be completed prior to the timed assessment. Standing balance will be assessed by the tandem stance as reported in the Short Physical Performance Battery [37]. Participants will be timed (up to 10 s) to stand in three different positions: feet side-by-side, semi-tandem stance, and tandem stance. Motor impairment will be assessed using the modified Fugl-Meyer (lower extremity) that was adapted for videoconferencing [38, 39]. The modified Fugl-Meyer (lower extremity) includes four tasks that are completed in a seated position (flexor synergy, extensor synergy, combined synergies, coordination and speed) [38, 39].

Fatigue will be measured using the nine-question Fatigue Severity Scale, which assesses the impacts of fatigue over the last week using an 8-point Likert scale (0 = strongly disagree, 7 = strongly agree) [40]. Stroke-related quality of life will be assessed using the Stroke Impact Scale (SIS), which comprises 8 domains focusing on (1) activities of daily living, (2) communication, (3) emotion, (4) hand function, (5) memory, (6) mobility, (7) participation, and (8) strength [41]. Health-related quality of life will be assessed using the five-question EuroQol-5D-5L (mobility, self-care, usual activities, pain/discomfort, anxiety/depression), which also includes a visual analogue scale to record a participant’s self-rated health today (0% = worst health, 100% = best health) [42]. The Patient Health Questionnaire-4 (PHQ-4) will evaluate symptoms of anxiety and depression over the last 2 weeks using a 4-point Likert scale (0 = “Not at all”, 3 = “Nearly every day”) [43]. Balance confidence will be assessed with the Activities-specific Balance Confidence (ABC) scale that evaluates a participant’s confidence in performing 16 activities while maintaining standing balance [44]. As exercise can improve cognitive function after stroke [45], cognitive function will be measured using the Montreal Cognitive Assessment Scale BLIND (MoCA-BLIND) [46]. The MoCA BLIND was adapted for remote and telephone delivery by excluding the visual and drawing tasks yet still evaluating abstraction, attention, delayed recall, language, memory, and orientation. All questionnaires will be read aloud by the assessor and screenshared for participants to see, except for the MoCA-BLIND.

As exercise may improve cardiovascular and metabolic risk factors for stroke [47], blood biomarkers will include fasting glucose, Haemoglobin A1c (HbA1c), Low-Density Lipoprotein (LDL), High-Density Lipoprotein (HDL), total cholesterol, and triglycerides. Participants will be asked to fast for at least 12 hours before attending a local blood collection facility.

Adherence will be assessed by recording the percentage of sessions attended by participants and the intensity of exercise achieved (using HRR). Protocol deviations will be tracked. Safety will be monitored through the number of falls and adverse and serious adverse events reported. Serious adverse events will be defined as admission to acute care.

### Data collection and monitoring

Data will be directly inputted into a database created using the Research Electronic Data capture (REDCap) system and housed on the University of British Columbia REDCap server. All data inputted into REDCap will be site-specific and de-identified except for participant demographics such as year of birth and sex. Each site will maintain access to site-specific data through REDCap. Site coordinators will meet weekly to discuss study progress, trial deviations, and recruitment strategies. Any unfavourable or unintended symptoms or events (e.g., pain or soreness from intervention, falls) that occur to participants during the trial will be reported as an adverse event in REDCap. All minor adverse events will be reported to the data safety monitoring board (DSMB) every 4 months and will be classified on severity and potential relatedness to the trial. Instructors will modify or stop exercises if minor adverse events occur during exercise sessions. Serious adverse events will be reported directly to the DSMB within 48 hours and according to site-specific ethics protocols.

A fidelity check will be completed for assessments and exercise sessions by the central team (primary coordinator) at regular intervals to ensure the study protocols are followed consistently across sites. The fidelity check will focus on how session expectations are communicated to participants, how sessions are managed, how exercises are progressed, ensuring participant safety, and ensuring a supportive environment for participants is created. Feedback will be provided immediately to assessors and instructors if any deviations to the protocol occur.

### Sample size estimates

In individuals living with chronic stroke, the minimal detectable change in 30 s STS score ranges from 1.21 [36] to 3.23 repetitions [35] with the standard deviation ranging from 3.66 to 3.86 [35, 36]. Task-specific training has been shown to improve 30 s STS score in those with chronic stroke by an average of 2.1 repetitions post-intervention [48]. Thus, using STATA (StataCorp LLC, Version 14.2, USA), a sample of 86 participants are required to detect a minimal detectable change of 2.1 repetitions, with a standard deviation of 3.86, power of 0.80, significance of 0.05, and controlling for baseline correlations (0.45; based on data from Liu-Ambrose et al. [45]). Anticipating an approximate 15% dropout rate, we will recruit a total of 100 participants (50 per group).

### Data analyses

Descriptive statistics (mean and standard deviation, median and interquartile range for continuous variables, and frequency and percentages for categorical variables) will be used to summarize all demographic and outcome variables. Linear mixed models will be used to examine the relationship between group assignment in 30 s STS score (without the use of arms) controlling for baseline scores in the outcome. The independent variables included in all models will be group assignment, time point, and an interaction between group and time. This approach includes all participants with an observation at either baseline or 10 weeks post-intervention in the intention to treat analysis. A sensitivity analysis using multiple imputation to address any missing data will also be run.

Secondary outcomes will be analysed using linear mixed models to determine the relationships between group assignments, controlling for baseline scores in the outcome. As assessments and interventions will be delivered remotely and across geographical locations (with overlapping regions), site will not be a variable in the model.

## Discussion

Delivering exercises through videoconferencing may increase accessibility to community programs and rehabilitation services in those discharged home after stroke. Although exercise interventions can improve mobility [23, 49] and may reduce the risk of falls [50] in people with stroke, therapists report challenges and safety barriers to treating mobility and balance over videoconferencing [20] and trials often exclude participants who do not have in-person assistance available [19, 21].

Seated exercise may be a safe alternative to traditional standing and walking exercise when offered remotely, as it can increase the stability of the exercising position, which may lower the risk of falls, therefore, not requiring in-person assistance. Evidence has also shown that seated exercises can transfer to improved mobility and balance in those living with stroke [22] and in older adults [51]. Dean et al. [52] found that 2 weeks of seated training (reaching tasks and lower limb loading) improved sitting and standing quality (reach distance and vertical force of paretic leg) in those with stroke. We recently reported, in a case-report, that 2 weeks of seated exercises that included task specific training (e.g., seated marching) improved balance, walking speed, and cardiorespiratory fitness in a person living with a chronic stroke [23].

Therefore, seated exercises may provide therapists the opportunity to safely treat mobility and balance impairments over videoconferencing in those with chronic stroke and mobility impairments. This further provides the opportunity to increase home-based rehabilitation, particularly for those living in rural regions that have limited access to community and rehabilitation programs.

### Limitations

Potential limitations such as internet speed, sufficient space within the home, and environment may impact interpretation of outcomes by assessors when conducted online. Lastly, as in-person assistance is not required during exercise sessions, instructors may limit exercise progressions if participant safety is a concern. This could result in a lower exercise intensity that may impact study results.

### Trial Status

Trial recruitment began on October 11th, 2023, at the central site and is expected to complete by December 1st, 2026. As of July 15th, 2025, 57 participants have been randomized into the study across all active sites. Study protocol version and date: version 9, dated April 5th, 2024.

## Supplementary Information

Supplementary Material 1.

Supplementary Material 2.

## Data Availability

All de-identified data will be made available in a public repository. Primary and secondary outcomes will be submitted for publication.

## Abbreviations

V-PASE: Virtual Physical Activity Seated Exercise Program
MSB: Minimum Sufficient Balance
HRR: Heart Rate Reserve
RPE: Rating of Perceived Exertion
STS: Sit-To-Stand
TUG: Timed Up and Go
EQ-5D-5L: EuroQol-5D-5L
PHQ-4: Patient Health Questionnaire-4
ABC: Activities-specific Balance Confidence
MoCA-BLIND: Montreal Cognitive Assessment BLIND
HbA1c: Haemoglobin A1c
LDL: Low-Density Lipoprotein
HDL: High-Density Lipoprotein
REDCap: Research Electronic Data Capture
DSMB: Data Safety Monitoring Board
