# Supplementary material for: Transferability of a 10-week remotely delivered Virtual Physical Activity Seated Exercise (V-PASE) program on post-stroke functional mobility: study protocol for a multisite randomized controlled trial
Source: Trials. 2026 Feb 6;27:198. doi: 10.1186/s13063-026-09523-8 (PMC12973678; doi:10.1186/s13063-026-09523-8)
Supplement: Supplementary file 1 — Supplementary Material 1. [file 13063_2026_9523_MOESM1_ESM.docx]

**Supplementary material**

Supplementary Table 1: V-PASE exercises

| **Exercises** | **Example session** |
| --- | --- |
| Air punching | 3 x 20 reps |
| Knee extensions | 3 x 20 reps |
| Seated kick boxing | 3 x 20 reps |
| Chair push-ups | 3 x 20 reps |
| Scooting forward and backwards on chair | 3 x 60s |
| Shoulders raises (lateral) | 3 x 20 reps |
| Mini lifts | 3 x 10 reps |
| Toe and heel raises | 3 x 20 reps |
| Seated jumping jacks | 3 x 20 reps |
| Reaching tasks beyond arm’s length | 3 x 20 reps |
| Abdominal crunches | 3 x 20 reps |
| Seated marching | 3 x 60s |
| Reps, repetitions; s, seconds | |
